# Supplementary material for: TNFα drives mitochondrial stress in POMC neurons in obesity
Source: Nat Commun. 2017 May 10;8:15143. doi: 10.1038/ncomms15143 (PMC5436136; doi:10.1038/ncomms15143)
Supplement: Supplementary Information — Supplementary Table and Supplementary Figures [file ncomms15143-s1.pdf]

Supplementary Table 1

Gene clone ID for ShRNA-mediated gene silencing TNFα downstream signals in *in vitro*

Symbol	Gene ID	RefSeqID	Clone ID
ATP6v1e2	74915	NM_029121	TRCN0000103700
			TRCN0000103701
			TRCN0000103702
			TRCN0000103703
			TRCN0000103704
BIRC2	11797	NM_007465	TRCN0000012303
			TRCN0000012304
			TRCN0000012305
			TRCN0000012306
			TRCN0000012307
CD40	21939	NM_011611	TRCN0000066243
			TRCN0000066244
			TRCN0000066245
			TRCN0000066246
Cox6a2	12862	NM_009943	TRCN0000076809
			TRCN0000076810
			TRCN0000076811
			TRCN0000076812
IKKβ	16150	NM_010546	TRCN0000026867
			TRCN0000026891
			TRCN0000026894
			TRCN0000026913
			TRCN0000026945
Map2k6 (MKK6)	26399	NM_011943	TRCN0000025259
			TRCN0000025260
			TRCN0000025261
			TRCN0000025262
			TRCN0000025263
Ndufab1	70316	NM_028177	TRCN0000041878
			TRCN0000041879
			TRCN0000041880
			TRCN0000041881
			TRCN0000041882
NFkB1	18033	NM_008689	TRCN0000009510
			TRCN0000009511
			TRCN0000009512
			TRCN0000009513
			TRCN0000009514
Park2 (Parkin)	50873	NM_016694	TRCN0000041143

45				TRCN0000041144
46				TRCN0000041145
47				TRCN0000041146
48				TRCN0000041447
49	PINK1	68943	NM_026880	TRCN0000026727
50				TRCN0000026733
51				TRCN0000026742
52				TRCN0000026743
53				TRCN0000026750
54	Stat3	20848	NM_011486	TRCN0000071453
55				TRCN0000071454
56				TRCN0000071455
57				TRCN0000071456
58				TRCN0000071457
59	Nr2c2 (TAK1)	22026	XM_132700	TRCN0000027044
60				TRCN0000027072
61				TRCN0000027073
62				TRCN0000027089
63	TNFrsf1a	21937	NM_011609	TRCN0000066103
64				TRCN0000066104
65				TRCN0000066105
66				TRCN0000066106
67				TRCN0000066107
68	TNFrsf1b	21938	NM_011610	TRCN0000012318
69				TRCN0000012319
70				TRCN0000012320
71				TRCN0000012321
72				TRCN0000012322
73	TNFrsf4	22163	NM_011659	TRCN0000066193
74				TRCN0000066194
75				TRCN0000066195
76				TRCN0000066196
77				TRCN0000066197
78	Tnfrsf6	18053	NM_033217	TRCN0000065553
79				TRCN0000065554
80				TRCN0000065555
81				TRCN0000065556
82				TRCN0000065557
83	TNFrsf10b	21933	NM_020275	TRCN0000012323
84				TRCN0000012324
85				TRCN0000012325
86				TRCN0000012326
87				TRCN0000012327
88	TNFrsf11a	21934	NM_009399	TRCN0000065698

89				TRCN0000065699
90				TRCN0000065700
91				TRCN0000065701
92				TRCN0000065702
93	TNFrsf11b	18383	NM_008764	TRCN0000065748
94				TRCN0000065749
95				TRCN0000065750
96				TRCN0000065751
97				TRCN0000065752
98	TNFrsf12a	27279	NM_013749	TRCN0000066883
99				TRCN0000066884
100				TRCN0000066885
101				TRCN0000066886
102				TRCN0000066887
103	TNFrsf13c	72049	NM_028075	TRCN0000066733
104				TRCN0000066734
105				TRCN0000066735
106				TRCN0000066736
107				TRCN0000066737
108	TNFrsf14	230979	NM_178931	TRCN0000065853
109				TRCN0000065854
110				TRCN0000065855
111				TRCN0000065856
112				TRCN0000065857
113	Tnfrsf16	14102	NM_007987	TRCN0000012328
114				TRCN0000012329
115				TRCN0000012330
116				TRCN0000012331
117				TRCN0000012332
118	TNFrsf17	21935	NM_011608	TRCN0000065898
119				TRCN0000065899
120				TRCN0000065900
121				TRCN0000065901
122				TRCN0000065902
123	TNFrsf18	21936	NM_009400	TRCN0000065953
124				TRCN0000065954
125				TRCN0000065955
126				TRCN0000065956
127				TRCN0000065957
128	TNFrsf21	94185	NM_052975	TRCN0000066598
129				TRCN0000066599
130				TRCN0000066600
131				TRCN0000066601
132				TRCN0000066602

TRADD	71609	NM_001033161	TRCN0000178874
			TRCN0000179091
			TRCN0000183578
			TRCN0000184240
TRAF2	22030	NM_009422	TRCN0000077228
			TRCN0000077229
			TRCN0000077230
			TRCN0000077231
			TRCN0000077232
TRAF5	22033	NM_011633	TRCN0000040718
			TRCN0000040719
			TRCN0000040720
			TRCN0000040721
			TRCN0000040722
TRAF6	22034	NM_009424	TRCN0000040733
			TRCN0000040734
			TRCN0000040735
			TRCN0000040736
			TRCN0000040737
TRAP1	68015	NM_026508	TRCN0000112170
			TRCN0000112171
			TRCN0000112172
			TRCN0000112173
			TRCN0000112174

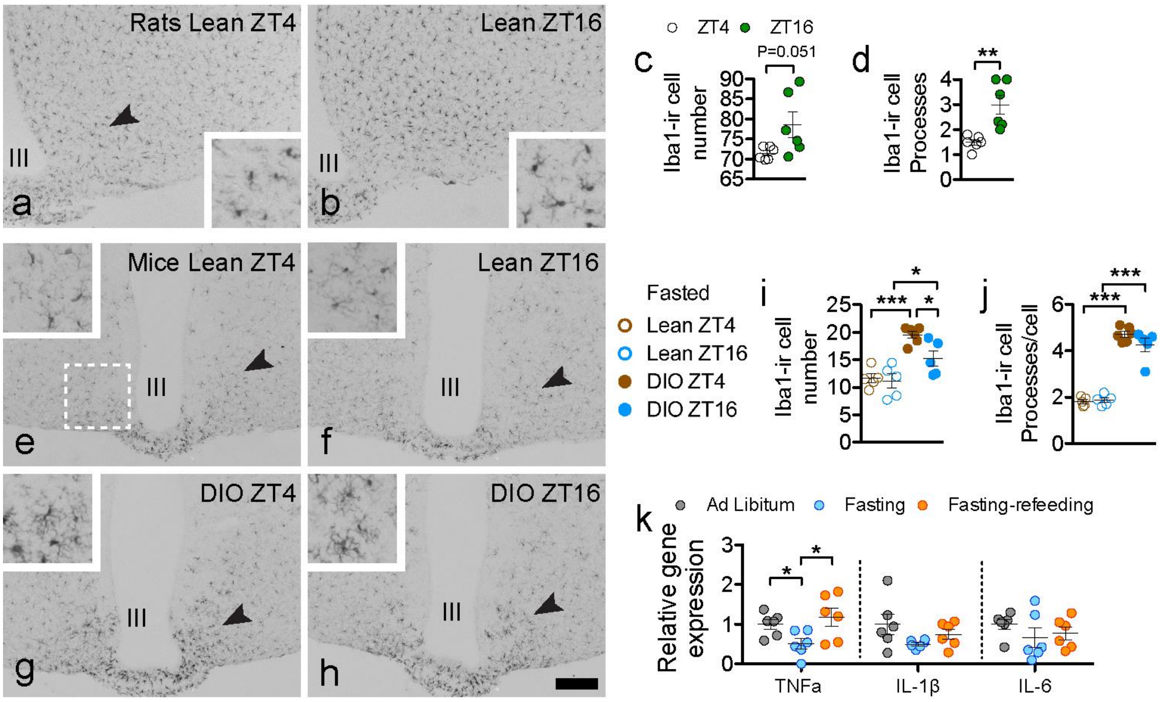

Supplementary Figure 1. The diurnal microglial activity patterns observed in chow-fed lean mice can be also observed in the MBH of rats on a chow diet, and fasting eliminates this diurnal pattern. (a-d) In chow-fed rats, although the difference in iba1-ir cell number did not reach significance between ZT4 and ZT16 (n=6 for ZT4 and ZT16, p=0.051), the number of iba1-ir cell processes in the MBH at ZT16 was significantly higher than at ZT4 (p=0.003). (e-h) Illustration of the iba1-ir cells in 24-h fasted lean or DIO mice. Cell number and processes per cell were quantified in a 0.2x0.2-mm frame outlined by dashed lines in e, high magnification of the cells pointed at by dark arrows is presented at the left-upper corner of each picture. (i and j) Fasting for 24 h eliminated the interaction between time and body weight on iba1-ir cell number and the number of processes in lean and DIO mice, there was an effect of body weight at ZT4 and ZT16 (n=5 for ZT4 and ZT16 of lean mice, n=6 for ZT4 and n=5 for ZT16 of DIO, for effect of bodyweight on iba1-ir cell number, $F(1,17)=32.76$, $p<0.0001$; for effect of body weight on iba1-ir cell processes, $F(1,17)=241.9$, $p<0.0001$), and there was an effect of time in DIO mice, $t_{10}=2.962$, $p<0.05$). (k) Fasting down-regulated, while refeeding stimulated, TNFα gene expression in the MBH of lean mice ($F(1,15)=4.377$, $p=0.032$). Scale bar: 160 µm in a, b, 200 µm in e-h. III: 3rd cerebral ventricle. *P<0.05, **P<0.01, ***P<0.001. Data are presented as means ± s.e.m. *P* values were analyzed by two-tailed Student's *t* test for unpaired comparisons in c, d, by two-way ANOVA followed by Bonferroni multiple comparisons in i, j; and by one-way ANOVA followed by post hoc *t* test in k.

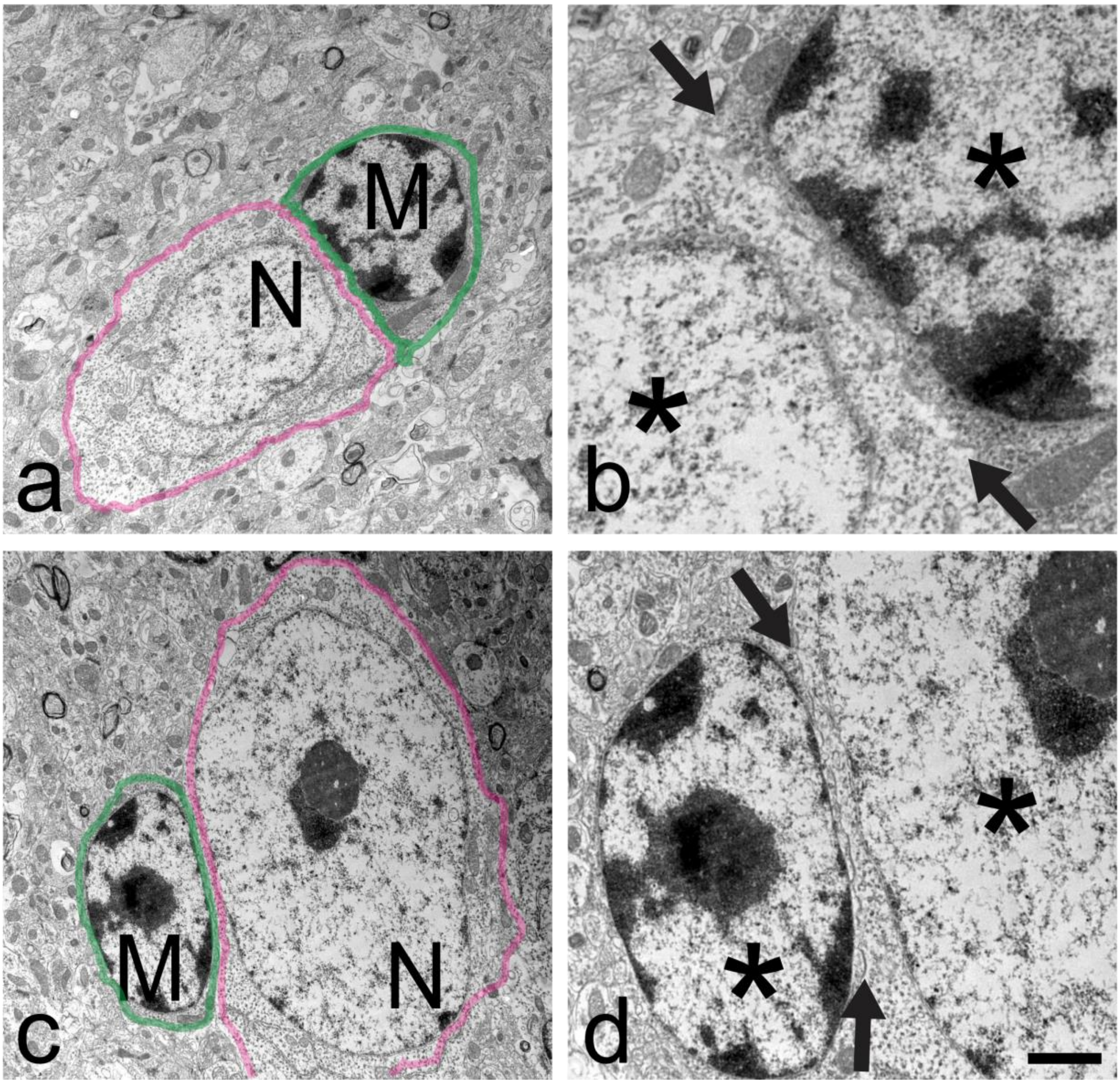

Supplementary Figure 2. Illustrations of the ultrastructure of microglial-neuronal soma very close contact in the hypothalamic arcuate nucleus (M: microglia, outlined in green, N: neuron, outlined in orange; *: nucleus of each cell). Arrows point to the contacting areas. Scale bar: 2μm in a and c, 1μm in b and d.

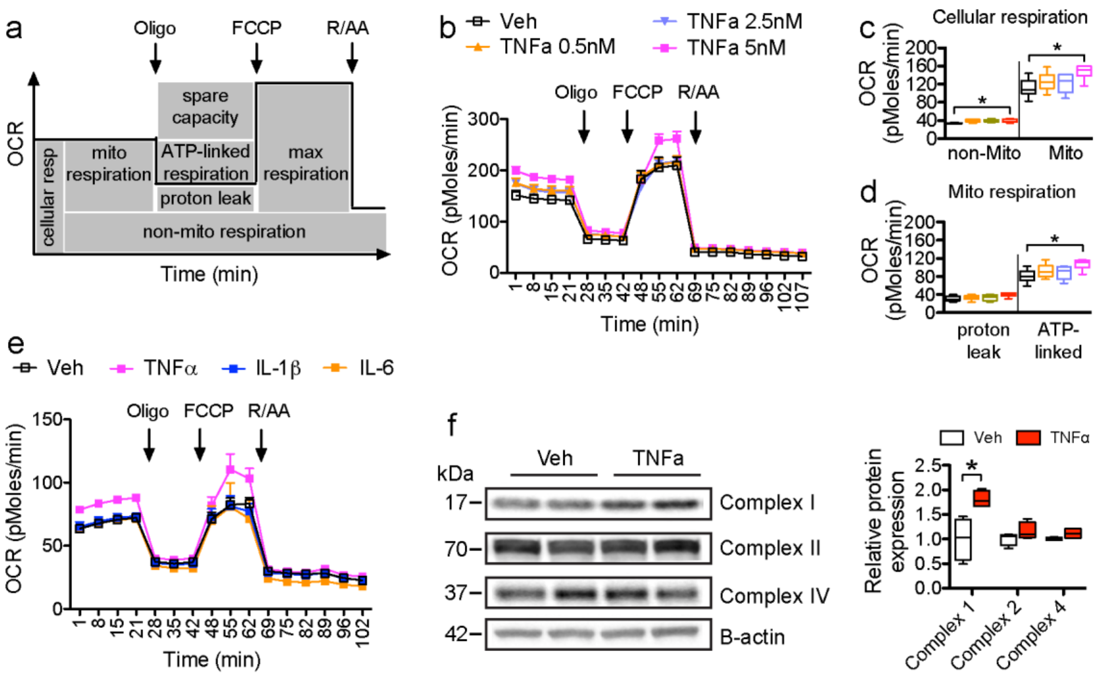

Supplementary Figure 3. TNFα stimulates hypothalamic neuronal mitochondrial activity. (a) Scheme depicting dissection of the oxygen-consuming processes of neurons. Cellular respiration was divided into mitochondrial and non-mitochondrial respiration using the ETC inhibitors rotenone (R) and antimycin A (AA). Mitochondrial respiration was further dissected into proton-leak and ATP-linked respiration using the ATP-synthase inhibitor oligomycin (Oligo). Maximal respiration was obtained after addition of an uncoupler (FCCP) and by subtracting the nonmitochondrial respiration rates. The portion of spare respiratory capacity was obtained by subtracting basal respiration from maximal respiration rates. Mitochondrial coupling efficiencies were calculated as the fraction of mitochondrial oxygen consumption that is sensitive to oligomycin, reflecting the fraction used to drive ATP synthesis. (b) Dose response of oxygen consuming rate (OCR) over time in primary hypothalamic neurons in response to different concentrations of TNFα. (c-d) Neurons treated by 5 nM TNFα had a significantly higher mitochondrial and ATP-linked oxygen consumption rate in comparison to those treated by vehicle, 0.5 nM or 2.5 nM TNFα. In addition, neurons treated with 5 nM TNFα also had a significantly higher non-mitochondrial OCR. (e) OCR over time in primary hypothalamic neurons treated with TNFα, IL-1β, IL-6, or vehicle. (f) Hypothalamic neurons treated with TNFα had significantly increased protein expression of complex-I, but not complex-II, or complex-IV (n=4 for each group, p=0.012 for complex-I). *P<0.05. Data are presented as means ± s.e.m in b, and min to max in d. *P* values were analyzed by Student's *t* test. See also Supplementary Fig 10.

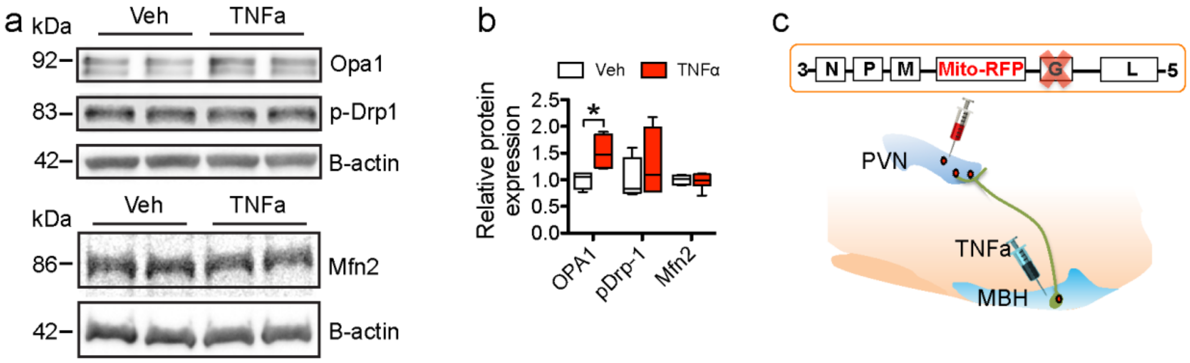

Supplementary Figure 4. TNFα stimulates hypothalamic neuronal mitochondrial fusion processes. Pseudotyped rabies virus (RABVΔG) was produced to analyze mitochondrial fusion-fission processes in *in vivo*. (a and b) Hypothalamic neurons treated with TNFα had significantly increased protein expression of optic atrophy 1 (Opa1), but not of mito-fusion 2 (Mfn2) or the phosphorylated dynamin-related protein 1 (p-Drp1) (n=4 for each group in Opa1 and p-Drp1 and n=6 for each group in Mfn2, p=0.034 for Opa1), see also Supplementary Fig 11. (c) The construction of RABVΔG encoding for mitochondrially-targeted red fluorescence protein ($Mito^{RFP}$), and the schematic illustration of virus injection into the paraventriculer nuclei (PVN) and the TNFα injection into the mediobasal hypothalamus (MBH). *P<0.05. Data are presented as min to max in b. *P* values was analyzed by Student's *t* test.

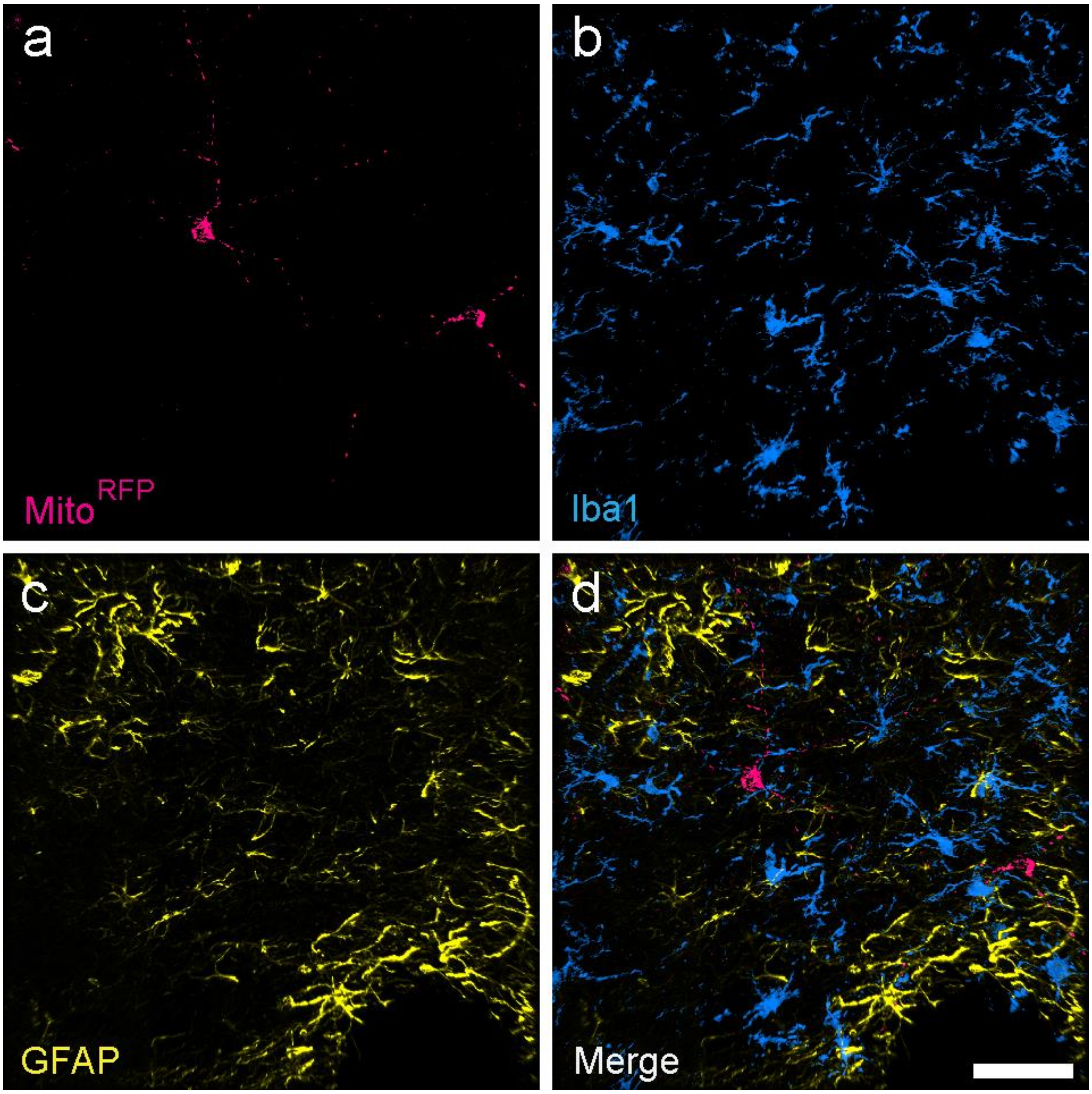

Supplementary Figure 5. Hypothalamic neurons infected by Mito^{RFP}-expressing RABVΔG did not stimulate immune responses from surrounding glial cells. (a) Hypothalamic neurons infected by Mito^{RFP}-expressing RABVΔG have Mito^{RFP}-labelled mitochondria. (b-d) No increase of iba1-ir microglia or GFAP-ir astrocytes occurred surrounding the PRV-infected neurons. Scale bar: 30 μm.

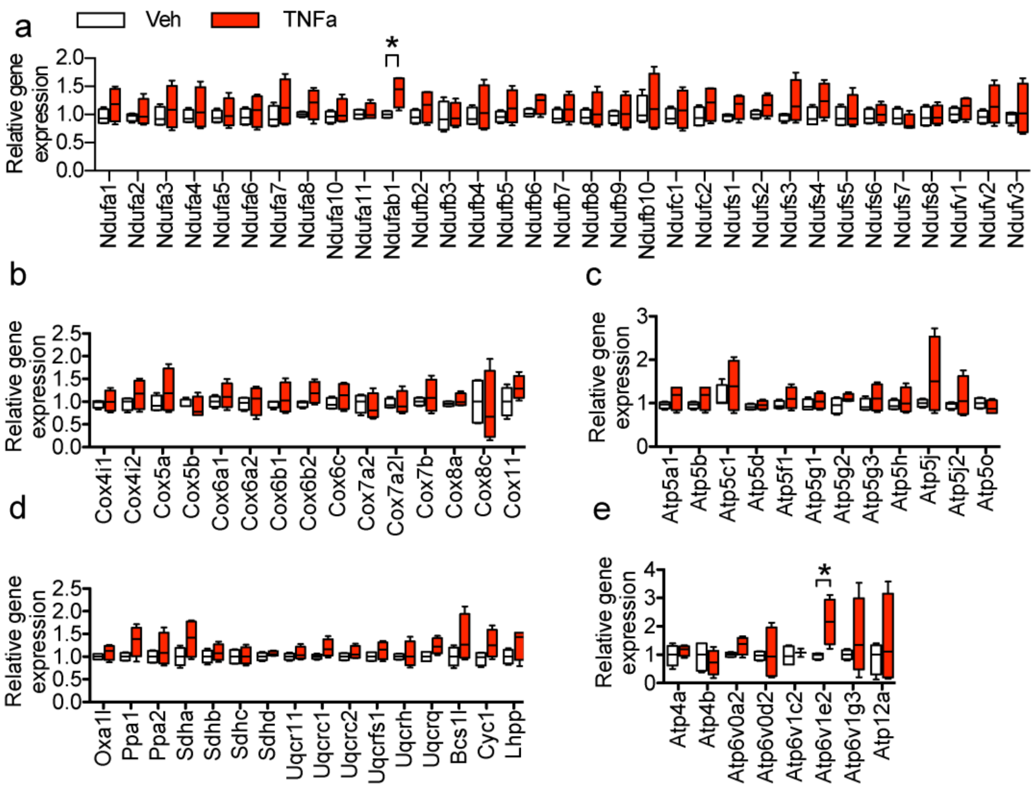

Supplementary Figure 6. Mitochondrial Energy Metabolism PCR Array in vehicle versus TNFα treatment in hypothalamic neurons. TNFα stimulated Atp6v1e2 (ATPase, H+ transporting, lysosomal V1 subunit E2) and Ndufab1 (NADH dehydrogenase (ubiquinone) 1, alpha/beta, subcomplex 1) gene expression (n=4 wells for vehicle and n=4 wells for TNFα, p=0.026 for Atp6v1e2, and p=0.031 for Ndufab1). NDUF: NADH dehydrogenase (ubiquinone), COX: Cytochrome c oxidase, ATP5: ATP synthase, Bcs1l: BCS1-like (yeast), Cyc1: Cytochrome c-1, Lhpp: Phospholysine phosphohistidine inorganic pyrophosphate phosphatase, Oxa1l: Oxidase assembly 1-like, Ppa: Pyrophosphatase (inorganic), Sdh: Succinate dehydrogenase complex, Uqcr: Ubiquinol-cytochrome c reductase. *$p<0.05$. Data are presented as min to max. *P* values were analyzed by Two-tailed Student's *t* test for comparing vehicle versus TNFα.

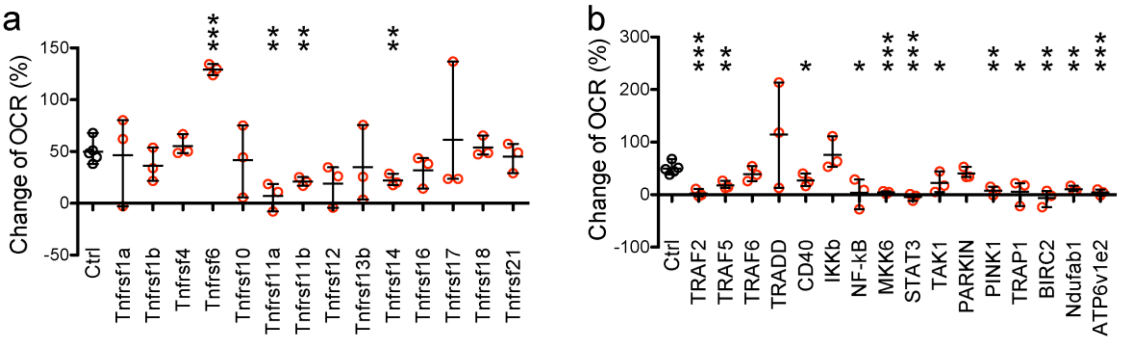

Supplementary Figure 7. Changes of cellular oxygen consuming rate (OCR) after knocking down each gene from the gene list of TNF receptors, the TNF receptor superfamily, and the TNF downstream-signalling pathway. (a) Changes of OCR with knocked down TNF receptors, and the TNF receptor superfamily ($F(1,36)=3.600$, $p=0.001$), knocked down Tnfrsf11a, 11b or 14 significantly attenuated the stimulatory effect of TNFα on OCR in comparison to Ctrl ($t_6=4.868$, $p=0.003$ for Tnfrsf11a, $t_6=4.219$, $p=0.006$ for Tnfrsf11b, $t_6=3.936$, $p=0.008$ for Tnfrsf14); knocking down Tnfrsf6 enhanced the stimulatory effect of TNFα on OCR ($p<0.001$). (b) Changes of OCR after knocking down the TNF downstream-signalling pathway ($F(1,36)=4.000$, $p=0.0003$); knocking down TNF receptor-associated factors 2 and 5 (TRAF2 and TRAF5, which mediate TNF effects on activation of MAPK8/JNK and NF-κB) significantly attenuates or completely blocks the TNF-stimulatory effects on OCR ($t_6=6.487$, $p=0.0006$ for TRAF2, and $t_6=4.340$, $p=0.0005$ for TRAF5). Among the rest of the TNF-downstream signals, knocking down cluster of differentiation (CD40), NF-κB, MAP kinase kinase 6 (MKK6), nuclear transcription factor signal transducer and activator of transcription 3 (STAT3, which can bind to mitochondrial DNA to regulate mitochondrial gene expression), the PTEN-induced putative kinase 1 (PINK1, mediating parkin protein-induced mitochondrial autophagy), TNF receptor-associated protein (TRAP1, a mitochondrial chaperone that regulates the metabolic switch between mitochondrial respiration and aerobic glycolysis), or apoptotic repression factor baculoviral inhibitors of apoptosis repeat-containing 1 (Birc1), can significantly attenuate or completely block the stimulatory effects of TNF on OCR ($t_6=6.487$, $p=0.034$ for CD40, $t_6=3.380$, $p=0.015$ for NF-κB, $t_6=6.778$, $p=0.0005$ for MKK6, $t_6=7.511$, $p=0.0003$ for STAT3, $t_6=2.580$, $p=0.042$ for TAK1, $t_6=5.686$, $p=0.001$ for PINK1, $t_6=3.619$, $p=0.011$ for TRAP1, $t_6=5.922$, $p=0.001$ for BIRC2). Furthermore, knocking down the selected mitochondrial targets, the mitochondrial respiratory chain complex-1 subunit - NADH dehydrogenase ubiquinone 1, alpha/beta subcomplex 1 (Ndufab1), or the V-type proton ATPase subunit e2 (ATP6v1e2), completely blocked the TNF-stimulatory effects on OCR ($t_6=5.594$, $p=0.001$ for Ndufab1, $t_6=6.470$, $p=0.0006$ for ATP6v1e2). *$P<0.05$, **$P<0.01$, ***$P<0.001$. Data are presented as means ± s.e.m. *P* values were analyzed by One-way ANOVA followed by post hoc *t* test.

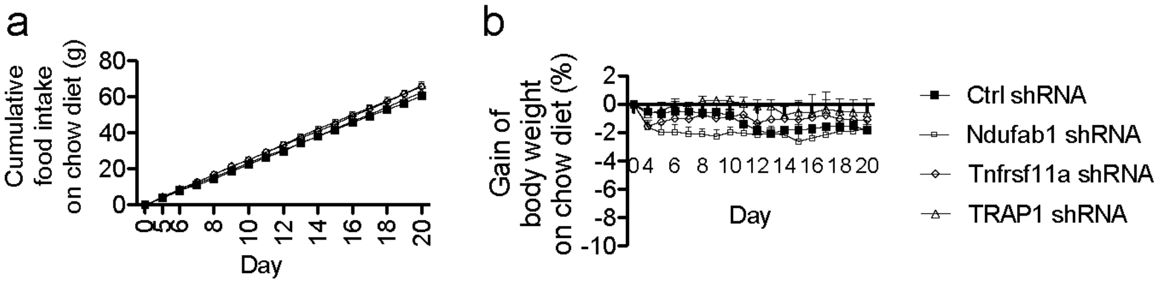

Supplementary Figure 8. Knocking down TNFα downstream signals by AAV-expressing Ndufab1 shRNA (n=5), Tnfrsf11a shRNA (n=4), or TRAP1 (n=4) in the MBH of chow-fed lean mice does not affect the food intake (a) or body weight gain (b), in comparison to mice that received AAV-expressing scrambled shRNA ($F(1,15)=1.665$, $p=0.217$ for food intake, and $F(1,15)=0.574$, $p=0.641$ for weight gain). Data are presented as means ± s.e.m. *P* values were analyzed by One-way ANOVA followed by post hoc *t* test for comparing daily food intake or bodyweight gain between scrambled shRNA group and each of the three-targeted shRNA groups.

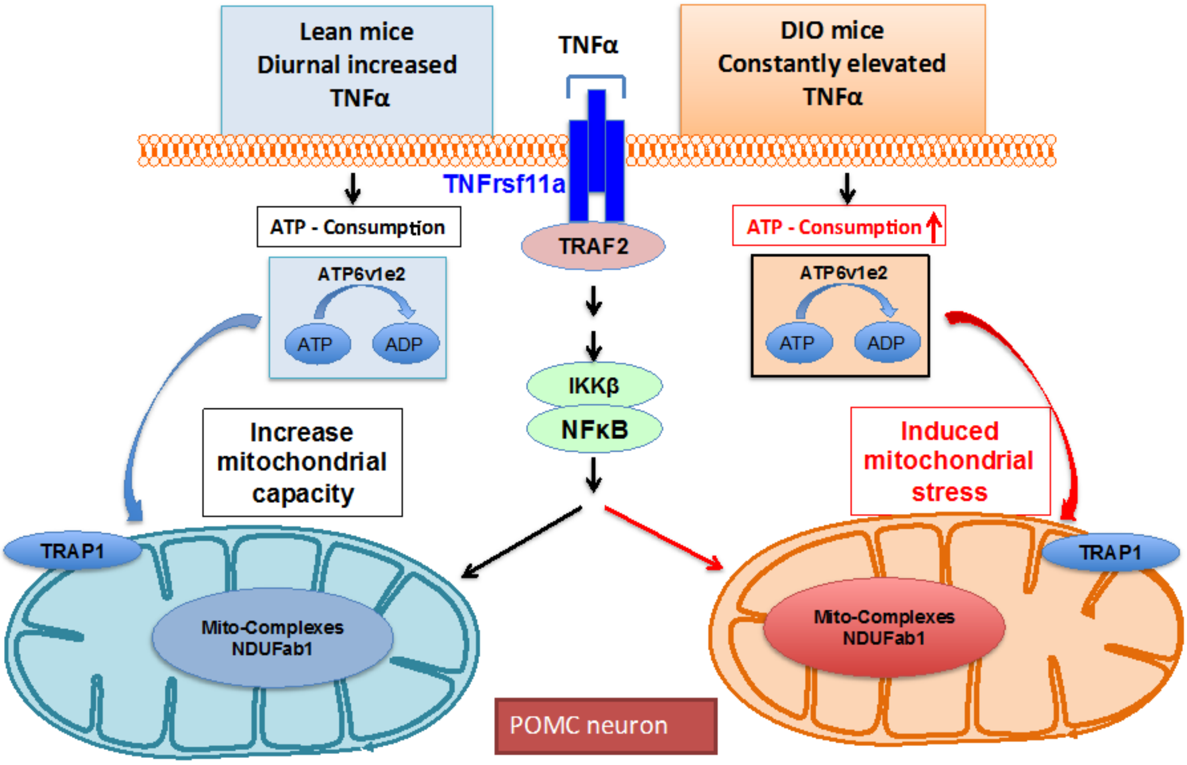

Supplementary Figure 9. In lean mice, TNFα engagement of TNFrsf11a activates NF-κB signaling pathway and enhances complex-1 activity to promote mitochondrial ATP production, consequently matching ATP production with demands in neurons in the dark phase. In DIO mice, constantly elevated TNFα drives persistent mitochondrial activity and causes mitochondrial stress in POMC neurons.

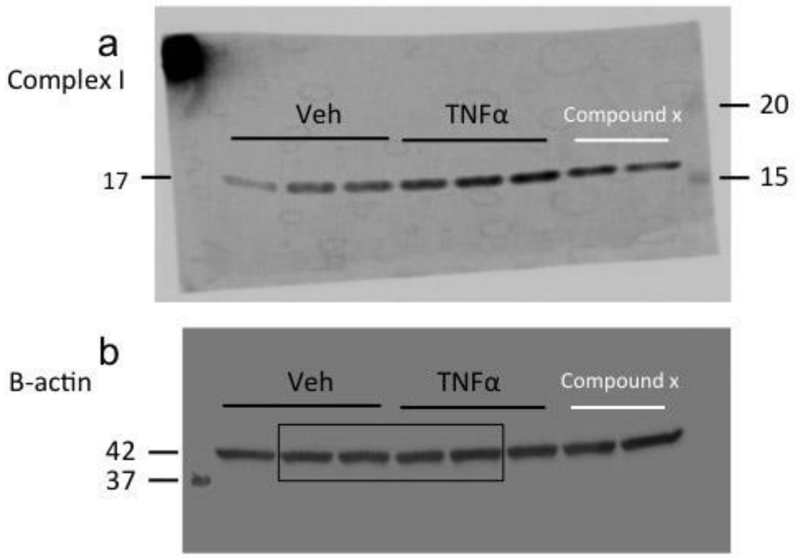

Supplementary Figure 10. Uncropped blots of Complex I (a) and B-actin (b) of supplementary figure 3f.

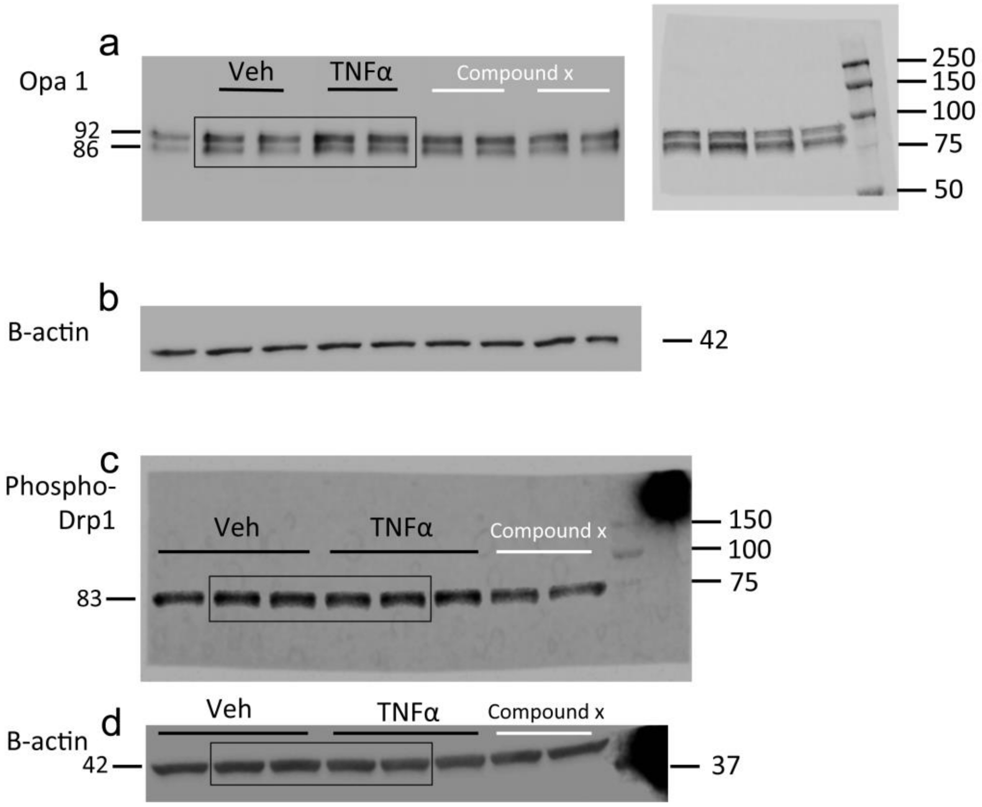

Supplementary Figure 11. Uncropped blots of Opa1 (a), p-Drp1 (c) and B-actin (d) of supplementary figure 4a. The image of the B-actin from Opa1 blot in b is not used in the figure. Instead, B-actin from p-Drp1 blot is presented).
